# HIV retention in care: results and lessons learned from the Positive Pathways Implementation Trial

**DOI:** 10.1186/s12875-022-01909-2

**Published:** 2022-11-23

**Authors:** Michael B. Wohlfeiler, Rachel Palmieri Weber, Laurence Brunet, Jennifer S. Fusco, Christine Uranaka, Quateka Cochran, Monica Palma, Tammeka Evans, Carl Millner, Gregory P. Fusco

**Affiliations:** 1 AIDS Healthcare Foundation, Los Angeles, CA USA; 2 Epividian Inc., Raleigh, NC USA; 3 ViiV Healthcare, Durham, NC USA

**Keywords:** HIV, HIV Treatment and Prevention, Retention in Care, Re-engagement, Alerts, Clinical Decision Support System

## Abstract

**Background:**

Sustained, routine care is vital to the health of people with HIV (PWH) and decreasing transmission of HIV. We evaluated whether the identification of PWH at-risk of falling out of care and prompts for outreach were effective in retaining PWH in care in the United States.

**Methods:**

In this cluster randomized controlled trial, 20 AIDS Healthcare Foundation Healthcare Centers (HCCs) were randomized to the intervention (*n* = 10) or control (*n* = 10) arm; all maintained existing retention efforts. The intervention included daily automated flags in CHORUS™, a mobile app and web-based reporting solution utilizing electronic health record data, that identified PWH at-risk of falling out of care to clinic staff. Among flagged PWH, the association between the intervention and visits after a flag was assessed using logistic regression models fit with generalized estimating equations (independent correlation structure) to account for clustering. To adjust for differences between HCCs, models included geographic region, number of PWH at HCC, and proportions of PWH who self-identified as Hispanic or had the Ryan White Program as a payer.

**Results:**

Of 15,875 PWH in care, 56% were flagged; 76% (intervention) and 75% (control) resulted in a visit, of which 76% were within 2 months of the flag. In adjusted analyses, flags had higher odds of being followed by a visit (odds ratio [OR]: 1.08, 95% confidence interval [CI]: 0.97, 1.21) or a visit within 2 months (OR: 1.07, 95% CI: 0.97, 1.17) at intervention than control HCCs. Among at-risk PWH with viral loads at baseline and study end, the proportion with < 50 copies/mL increased in both study arms, but more so at intervention (65% to 74%) than control (62% to 67%) HCCs.

**Conclusion:**

Despite challenges of the COVID-19 pandemic, adding an intervention to existing retention efforts, and the reality that behavior change takes time, PWH flagged as at-risk of falling out of care were marginally more likely to return for care at intervention than control HCCs and a greater proportion achieved undetectability. Sustained use of the retention module in CHORUS™ has the potential to streamline retention efforts, retain more PWH in care, and ultimately decrease transmission of HIV.

**Trial Registration:**

The study was first registered at Clinical Trials.gov (NCT04147832, https://clinicaltrials.gov/show/NCT04147832) on 01/11/2019.

**Supplementary Information:**

The online version contains supplementary material available at 10.1186/s12875-022-01909-2.

## Background

Sustained, routine HIV care is vital to the overall health of people with HIV (PWH). Indeed, engagement in routine HIV care for what has become a chronic disease due to the success of antiretroviral therapy has been associated with improved health outcomes [1, 2] and decreased mortality [1–7]. The benefits of being engaged and retained in care can also reach beyond the clinical health of individual PWH by ideally resulting in undetectable viral loads and decreased transmission of HIV in the community [5, 8–10]. Based on data from the National HIV Surveillance System and the Medical Monitoring Project, it was estimated that PWH who were undiagnosed or diagnosed but not retained in care were responsible for over 90% of the HIV transmissions in 2009 [9, 10]. By the end of 2019, it was estimated that there were almost 1.2 million PWH in the United States (US), 13% of whom were undiagnosed [11]. Sixty-six percent of PWH received HIV medical care in 2019 (i.e., ≥ 1 CD4 or viral load tests) and only 50% were retained in HIV medical care (i.e., ≥ 2 CD4 or viral load tests performed ≥ 3 months apart) [12].

The American Recovery and Reinvestment Act of 2009 [13], among other publications [14–20], prioritized the development and use of health information systems and electronic health records (EHR) in order to bridge the gap between evidence and practice. These reports asserted that evidence-based clinical decision support systems (CDSS) may be a strategy for improving the quality, safety, and cost of healthcare. Though some studies of CDSS have reported positive results, they have been focused on primary care, medication management, and diabetes care [21–31]; challenges including alert fatigue, suboptimal compliance, database inconsistencies, and unintended consequences have also been described [32–34]. However, a 2005 review of 100 randomized and nonrandomized controlled trials [35] found that CDSS did improve provider performance in 64% of studies, including 76% of studies that utilized reminder systems. Improved provider performance was associated with CDSS that automatically prompted users compared to CDSS that required the provider to activate the system (73% versus 47%, *p* = 0.02); however, the effect on patient outcomes was inconsistent or not reported [35]. The Virology Fast Track Study was a pivotal randomized controlled trial (RCT) that assessed the use of a CDSS in improving quality of care for PWH and informed the conceptualization and design of this study. Interactive alerts identifying adverse events and missed appointments were disseminated to each provider’s EHR home page, the patient specific EHR page, and in biweekly emails. Static alerts, which did not include scheduling capabilities, were only disseminated to the patient specific EHR page. The rate of 6-month suboptimal follow-up was lower, the mean increase in CD4 cell counts was higher, and the median time-to-next scheduled appointment after a missed appointment was lower in the interactive alert group than in the static alert group [36].

Identifying ways to retain PWH in routine HIV care may contribute to not only improving clinical outcomes for the individual but may also help meet national HIV goals of ending the HIV epidemic in the US by reducing transmission [10, 37, 38]. In the Positive Pathways Implementation Trial, we evaluated whether a CDSS utilizing alerts identifying PWH at-risk of falling out of care and prompts for enhanced contact (i.e., outreach) were effective in re-engaging PWH in care at AIDS Healthcare Foundation Healthcare Centers across the US.

## Methods

### Intervention

Clinical Health Outcomes Reporting & Utilization Service (CHORUS™) is a web portal and mobile application translating EHR data into meaningful information and actionable alerts for healthcare providers and clinic staff. Because CHORUS™ utilizes data already entered in the EHR, there’s no additional data entry or software to purchase. The intervention for the Positive Pathways study consisted of daily automated alerts in the CHORUS™ Retention in Care (RIC) Module warning of suboptimal clinic attendance by PWH active in care (i.e., PWH at-risk of falling out of care). Eligible PWH were aged ≥ 18 years and had received HIV care in the prior 13 months (i.e., visit with healthcare provider or HIV lab measurement).

**Fig. 1:**
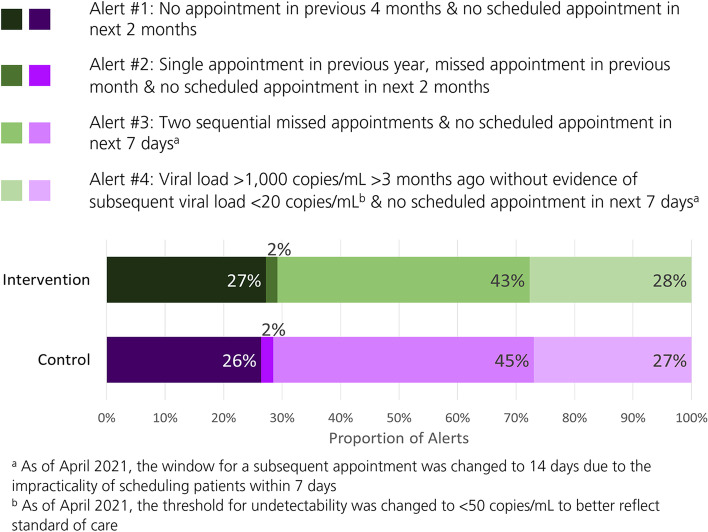
Alert Types and Distribution Over Follow-Up Up Among Intervention HCCs (*n* = 8,860 alerts) and Control HCCs (*n* = 6,878 alerts)

Alerts were based on a combination of historical factors (i.e., timing of last appointment, missed appointments, or a viral load of > 1,000 copies/mL more than 3 months in the past) and timing of future appointments (i.e., in the next 2 months or 14 days) (Fig. 1). Eligible PWH had the potential to meet criteria for more than one type of alert on the same day. To identify PWH at-risk of falling out of care more broadly, flags identified PWH who met criteria for ≥ 1 alert over a consecutive period. Healthcare providers and clinic staff were prompted to re-engage PWH at-risk of falling out of care and to schedule an appointment; recording the outcomes of their outreach efforts in CHORUS™ was encouraged.

### Study design

The Positive Pathways Implementation Trial was conducted at AIDS Healthcare Foundation (AHF), a global nonprofit organization that has provided HIV/AIDS medical care at Healthcare Centers (HCCs) to over 1.6 million people in 45 countries. Twenty AHF HCCs in the US were randomly selected to participate in this parallel, cluster RCT and subsequently randomized to either the control or intervention arms of the study. The control HCCs maintained the existing AHF-wide retention effort throughout the study that included a monthly list of PWH who had been out of care for ≥ 104 days [39]. PWH identified as at-risk of falling out of care in this study were documented in the study dataset but were not made known to clinic staff at the control HCCs. The intervention HCCs maintained the AHF-wide existing retention effort and additionally received the study intervention via the CHORUS™ RIC Module. Alerts were recorded from October 2020 through May 2021; PWH were followed through July 2021.

### Statistical analyses

Baseline characteristics of the AHF HCCs, as well as alerts, flags, appointments, and visits over the study period, were described for the intervention and control arms of the study. Data on alerts and flags were obtained directly from CHORUS™; all other data were obtained from the Observational Pharmaco-Epidemiology Research & Analysis (OPERA^®^) longitudinal healthcare database of prospectively captured clinical data from the EHRs. The primary outcome of interest was re-engagement, defined as a completed visit (a) any time over follow-up or (b) ≤ 2 months after being flagged. Visits were any contact with the HCC including an in-person or telehealth visit with a provider, prescription refill request, or lab draw. Among PWH who were flagged, the association between the intervention and re-engagement was assessed using logistic regression models fit with generalized estimating equations (independent correlation structure) to account for correlations within the clusters (i.e., AHF HCCs). To adjust for differences between the HCCs, models were adjusted for the following HCC characteristics: geographic region (Northeast, South, Midwest, West), size (i.e., number of PWH active in care), proportion of active PWH who self-identified as Hispanic/Latino, and proportion of active PWH with the AIDS Drug Assistance Program (ADAP)/Ryan White Program as a payer. Among PWH who were retained in care at both the control and intervention HCCs, we described HIV viral load at baseline and end of follow-up.

### Implementation feedback

We sought feedback from AHF workforce members at the intervention HCCs via focus groups near the conclusion of the study, weekly meetings with AHF leadership, and surveys. Surveys were disseminated to AHF workforce members through the CHORUS™ mobile application after first usage, within 30 days of use, and after 3, 6, and 9 months of use. Each survey included 5–12 questions related to the acceptability, adoption, and sustainability of the intervention within the clinic, and whether the intervention identified and solved a problem related to patients falling out of care.

## Results

At the time of randomization, 38 of 64 AHF HCCs were eligible to be selected for the study; reasons for exclusion included non-US location, small size (i.e., < 100 PWH active in care at location), satellite locations to other HCCs, and participation in pilot activities. Twenty HCCs were randomly selected to the study and subsequently randomized to the control (*n* = 10) or intervention (*n* = 10) arms of the study. The intervention HCCs were more likely to be in the Southern US, care for more PWH, and employ more healthcare providers; a greater proportion of PWH at the intervention HCCs reported the ADAP/Ryan White Program as a payer (Table 1).

**Table 1:** Characteristics of Participating AIDS Healthcare Foundation Healthcare Centers

	Intervention	Control
Total HCC per arm, N	10	10
HCCs in Southern US, n	7	5
Healthcare providers per HCC, median (IQR)	10 (4, 22)	8 (5, 11)
Total PWH per arm, N	8,836	7,039
PWH per HCC, median (IQR)	1081 (621, 1812)	1018 (559, 1649)
PWH with Hispanic/Latino ethnicity per HCC, median percentage (IQR)	18 (7, 34)	20 (9, 23)
PWH with the ADAP/Ryan White Program as a payer per HCC, median percentage (IQR)	36 (23, 68)	28 (19, 42)
PWH with HIV viral load < 200 copies/mL, median percentage (IQR)	88 (86, 90)	87 (85, 92)
PWH with any mental health disordera, median percentage (IQR)	8 (7,11)	11 (7, 13)

*ADAP* AIDS Drug Assistance Program,* IQR* Interquartile range, *N* Number, *PWH* People with HIV

^a^Includes anxiety disorders, bipolar or manic disorders, major depressive disorder, schizophrenic disorder, dementia, suicidality

There were 15,875 PWH who were active in care (8,836 at intervention HCCs; 7,039 PWH at control HCCs) and therefore, eligible to be identified as at-risk of falling out of care; overall, 56% were flagged at least once. A total of 15,738 alerts occurred during the study. PWH were most frequently identified as at-risk of falling out of care due to 2 missed appointments, without any scheduled appointment in the next 2 weeks (alert #3); this alert accounted for 44% of the total alerts. In contrast, alert #2 (i.e., single appointment in previous year, missed appointment in previous month, and no scheduled appointment in next 2 months) accounted for only 2% of the total alerts. The distribution of alert types was similar between the intervention and control arms (Fig. 1).

**Fig. 2:**
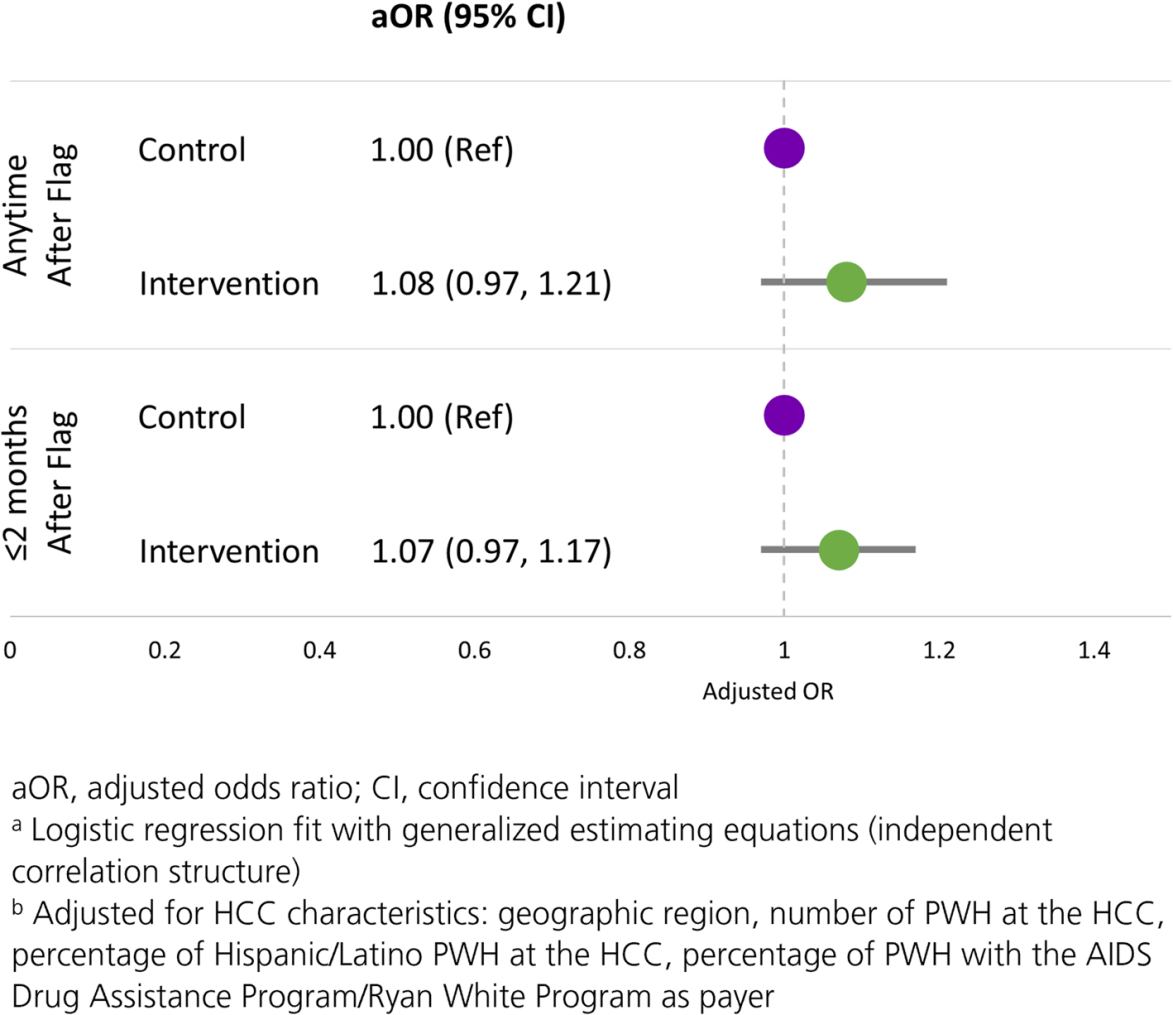
Adjusted Odds Ratios for the Association Between the Intervention and Visits After Flags

A total of 13,002 flags, representing a consecutive period of alerts, occurred during the study. At intervention HCCs, 56% of PWH were flagged at least once and 17% were flagged at least twice; a similar proportion of PWH at control HCCs were flagged (55% and 17%, respectively). In the intervention and control arms, 90% and 86% of flags resulted in an appointment while 76% and 75% resulted in a visit, respectively. At both intervention and control arm HCCs, appointments were scheduled for a median 4 days after a flag; visits occurred a median one month after a flag. Overall, 25% of visits were within 14 days and 76% were within 2 months of the flag; results were similar between the intervention and control arms (Table 2). In adjusted analyses, flags at intervention HCCs had 8% and 7% higher odds of being followed by a visit (adjusted odds ratio [aOR]: 1.08, 95% confidence interval [CI]: 0.97, 1.21) or a visit within 2 months (aOR: 1.07, 95% CI: 0.97, 1.17), respectively, than flags at control HCCs over the 10-month study period (Fig. 2).

**Table 2:** Completed Appointments and Visits After Flags

	Intervention	Control
Total number of flags	7,355	5,649
Flags with a subsequent appointment, n (%)	6,584 (90)	4,880 (86)
Days between flag and appointment, median (IQR)	4 (2, 16)	4 (2,15)
Flags with a subsequent visit, n (%)	5,580 (76)	4,249 (75)
Days between flag and visit, median (IQR)	32 (15, 60)	30 (12, 59)
Flags with a subsequent visit within ≤ 14 days, n (%)	1,361 (24)	1,093 (26)
Flags with a subsequent visit within ≤ 1 month, n (%)	2,668 (48)	2,127 (50)
Flags with a subsequent visit within ≤ 2 months, n (%)	4,200 (75)	3,246 (76)

*IQR* Interquartile range, *n* Number

Of the 4,977 PWH at intervention and 3,888 PWH at control HCCs who were identified as at-risk of falling out of care (i.e., they met criteria for ≥ 1 alert during the study), 59% (of PWH overall and in each study arm) returned to their HCC and had viral load measurements at both baseline and end of follow-up. The proportion of PWH suppressed to a viral load < 50 copies/mL increased by 9% at intervention HCCs (65% to 74%) compared to 5% at control HCCs (62% to 67%).

Finally, 98 AHF workforce members signed informed consent for study surveys to be sent to them in the CHORUS™ mobile application. A total of 15 unique workforce members completed ≥ 1 survey(s) over the course of the study, for a global response rate of 15%. Representatives from 7 intervention HCCs provided additional feedback during two focus groups in July 2021 just prior to study completion. Results from these efforts are available in Additional File 1.

## Discussion

More than 50% of the 15,875 PWH in care at 20 participating AHF HCCs were identified as at-risk of falling out of care. While most flags resulted in appointments (90% at intervention and 86% at control HCCs), approximately three quarters resulted in visits; most visits occurred within 2 months of the flags. After adjusting for clustering within HCCs and for differences between HCCs, the odds of flags being followed by visits were marginally higher (7–8%) at intervention than control HCCs. Finally, among at-risk PWH with viral loads at both baseline and study end, the proportion with viral load < 50 copies/mL increased in both study arms, but more so at intervention (65% to 74%) than control (62% to 67%) HCCs.

### Strengths of the study

One of the major strengths of this study’s intervention is that CHORUS™ utilizes existing EHR data such that no additional data collection was required and no software needed to be purchased such that any clinic, large or small, could participate. Additionally, the CDSS allowed for patient information to be at the fingertips of a clinic’s healthcare providers and staff members via both a mobile application and web portal. The CHORUS™ RIC Module was intuitive and easy to use; most survey answers and feedback from the focus groups were positive or neutral with respect to usability of the CDSS. Another strength of the study is that though there was a single healthcare/EHR system, which allowed for data consistency across the clinics, there was rich heterogeneity in the clinic populations, staff, and clinic operations across the geographically diverse HCCs. The entire workforce at intervention HCCs was trained on the CHORUS™ RIC Module and participated in discussions about how to best incorporate the intervention into their clinic’s workflow. Involving clinic staff at different levels of care had a positive impact on overall culture change within a HCC and engaged staff, regardless of their role at the HCC (e.g., healthcare provider, administrative), presented innovative ideas for reaching hard-to-contact PWH. The retention in care efforts were truly a team effort that allowed HCCs to determine workflows that worked for their unique workforces and patient populations. Finally, as reported by numerous people at HCCs and within AHF leadership, the biggest value of the study was to change the “one and done” mindset of sites that would make a singular call to a patient before giving up; clinics realized it takes multiple calls, sometimes from multiple people, to keep patients engaged. Indeed, weekly emails sent to intervention HCCs that summarized data already available in the CHORUS™ RIC Module helped to reinforce the idea that retention is an ongoing, continuous process.

### Challenges and limitations

This implementation trial faced substantial challenges. First, this study began just as the third wave of the global COVID-19 pandemic was hitting the US. This resulted in heightened pressures on the HCCs due to a greater demand for care from patients (COVID-19 testing, treatment, and vaccination), limited human resources resulting from COVID-19 infections among providers and clinic staff, and restrictive (but necessary) pandemic procedures limiting the number of available appointments. Clinic operations were additionally impacted by extreme weather events during the study (e.g., February 2021 winter storm and 2020 Hurricanes Delta, Zeta, and Eta). Second, this study was originally designed to compare the CHORUS™ RIC Module intervention with the standard of care retention effort at AHF. AHF leadership was reluctant to cease their established retention effort (i.e., the 104-day report) and as a result, the study intervention was added to the established workflow at HCCs in the intervention arm. There was some overlap between the study intervention and the standard AHF practice of contacting patients who had been out of care for ≥ 104 days. While the 104-day report identified patients without a recent visit, it did not factor in scheduled appointments, prior appointment patterns, or high viral load measurements as the alerts did in the CHORUS™ RIC module. Additionally, the non-interactive 104-day report was released monthly and only to the clinic manager; the CHORUS™ RIC module was refreshed daily and could be utilized by any provider or staff member at the HCC. Layering one intervention/workflow process on top of another overwhelmed staff and impacted clinic morale; streamlining the logistics of reporting and documenting within one electronic system linked to the EHR would have potentially freed up time and resources for more patient outreach and care. Though the CHORUS™ RIC Module was well-received by the AHF workforce who utilized it and preferred by many who participated in the focus groups, it was still duplicative.

Though there is clear benefit to using existing clinical data, there are also challenges that come from basing an implementation science study on EHR data. EHRs are complicated, not always kept up to date by providers and clinic staff, and information is sometimes entered in different fields than anticipated. Outdated or incorrect information in the EHR was carried over to the CHORUS™ RIC Module, sometimes resulting in inefficient and unnecessary work by clinic staff. For example, clinic staff discovered that 17 PWH flagged by alerts had died prior to the start of the study; though this is valuable information to have, it highlights the fact that clinics aren’t always engaged with all their patients. Additionally, the CDSS functions can be negatively impacted by disruptions caused by upgrades to the EHR system or technical difficulties. In this study, the extract, transform, load process was down for two weeks in December 2020, a month where AHF HCCs are known to already see a decrease in visits; this was a period in which the CDSS alerts would likely have been even more valuable to the HCCs.

Finally, there are additional limitations of the study design and intervention to consider. First, alerts identifying PWH at-risk of falling out of care could be resolved by either a scheduled appointment during the referenced timeframe (i.e., within 2 months or 14 days, depending on the specific alert) or a completed visit. This study showed that not all scheduled appointments resulted in a completed visit among individuals who were flagged as at-risk of falling out of care; outreach from the clinic likely needs to persist until, at the very least, PWH have completed a visit with a provider, a truer marker of retention. The short study timeline likely did not allow enough time for the intervention to be fully incorporated into clinic operations or for all flagged PWH to return to the clinic; behavior change, from both clinic workforce and patients, takes time. While this study showed a meaningful trend toward retention, the results did not reach statistical significance for a difference between the HCCs which received the intervention and those that did not. Additionally, alerts may need to be revised to better suit specific clinics and patient populations. As a result of weekly meetings with AHF leadership, alerts #3 and #4 were modified to reflect more reasonable timeframes in which appointments could be scheduled, especially under COVID-19 conditions, and what viral thresholds to use. Finally, it was challenging to assess clinical outcomes in this study as they could only be observed among PWH who were ultimately retained in care.

## Conclusion

Re-engaging PWH at-risk of falling out of care and retaining them in care is challenging. Despite the COVID-19 pandemic with a surging third wave during the study, an intervention that was layered on top of an existing retention report at a time when clinic resources were already depleted, and a short study duration of 8 to 10 months (among other challenges), flags that occurred at intervention HCCs were more likely than flags at control HCCs to result in a subsequent visit at any time over follow-up or within 2 months of the flag. Additionally, more of the at-risk PWH who returned to the HCC achieved undetectability at intervention HCCs than control HCCs during the study. Cross-discipline teamwork and resourcefulness at the intervention HCCs likely contributed to the positive clinical and retention outcomes. Re-engagement of PWH identified as at-risk of falling out of care is not the same as sustained retention-in-care and future studies should evaluate interventions that aim to improve both over a longer period of time. Behavior change takes time; the identification and re-engagement of PWH at risk of falling out of care cannot be a “one and done” situation. Sustained use of the CHORUS™ RIC Module has the potential to streamline retention efforts, retain more PWH in care, and ultimately decrease transmission of HIV.

## Supplementary Information

**Additional file 1.**

## Data Availability

The datasets used in this study are not publicly available due to privacy concerns and the proprietary nature of the database but can be accessed upon reasonable request through the corresponding author to the OPERA^®^ Epidemiology and Clinical Advisory Board. Access to codes may be granted upon request with parties agreeing to privacy restrictions and technological specifications and requirements.

## Abbreviations

ADAP: AIDS Drug Assistance Program
AHF: AIDS Healthcare Foundation
aOR: Adjusted Odds Ratio
CDSS: Clinical Decision Support System
CHORUS™: Clinical Health Outcomes Reporting & Utilization Service
CI: Confidence Interval
EHR: Electronic Health Record
HCC: Healthcare Center
IQR: Interquartile Range
N: Number
OPERA^®^: Observational Pharmaco-Epidemiology Research & Analysis
PWH: People with HIV
RCT: Randomized Controlled Trial
RIC: Retention in Care
US: United States
