# Supplementary material for: HIV retention in care: results and lessons learned from the Positive Pathways Implementation Trial
Source: BMC Prim Care. 2022 Nov 23;23:297. doi: 10.1186/s12875-022-01909-2 (PMC9685944; doi:10.1186/s12875-022-01909-2)
Supplement: Supplementary file 1 — Additional file 1. [file 12875_2022_1909_MOESM1_ESM.pdf]

HIV Retention in Care: Results and Lessons Learned from the Positive Pathways Implementation Trial

Additional File 1

Michael B. Wohlfeiler[1], Rachel Palmieri Weber[2*], Laurence Brunet[2], Jennifer S. Fusco[2], Christine Uranaka[1], Quateka Cochran[1], Monica Palma[1], Tammeka Evans[3], Carl Millner[1], Gregory P. Fusco[2]

[1] AIDS Healthcare Foundation, Los Angeles, CA, USA

[2] Epividian Inc., Raleigh, NC, USA

[3] ViiV Healthcare, Durham, NC, USA

*Corresponding Author:

Rachel Palmieri Weber

rachel.weber@epividian.com

Surveys via the CHORUS™ Retention in Care Module App

Ninety-eight AIDS Healthcare Foundation (AHF) workforce members signed informed consent for study surveys to be sent to them in the CHORUS™ mobile application. A total of 15 unique AHF workforce members completed ≥ 1 survey(s) over the course of the study, for a global response rate of 15% (**Additional Table 1**). Results by the following timepoints are presented below:

- After the first use: **Additional Figures 1** and **2**
- After 30 days: **Additional Figure 3**
- After 3 months: **Additional Figures 4** and **5**
- After 6 months: There were not enough respondent to provide results and maintain anonymity.
- After 9 months: There were not enough respondent to provide results and maintain anonymity.

Additional Table 1. Summary of Surveys Received Through the CHORUS™ Retention in Care Module App

Survey	Number of Survey Questions	Number of Surveys Completed
After the First Use	7	10
After 30 Days	5	7
After 3 Months	9	5
After 6 Months	12	2
After 9 Months	9	1
All Surveys	42	25

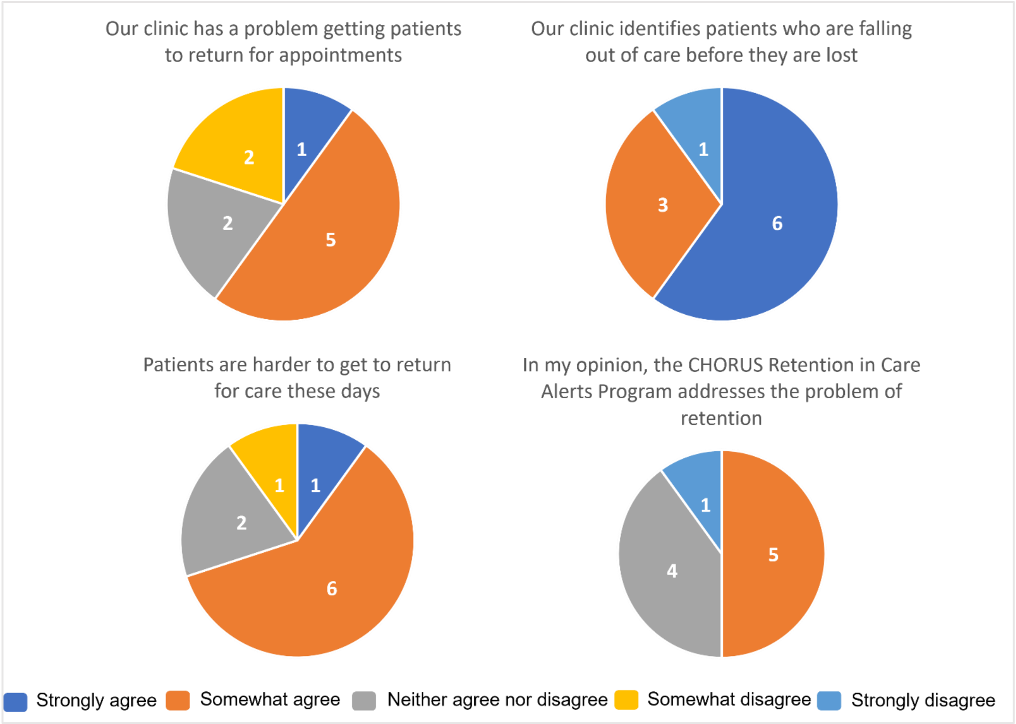

Additional Figure 1. Results of After First Use Survey: Appropriateness Domain Questions

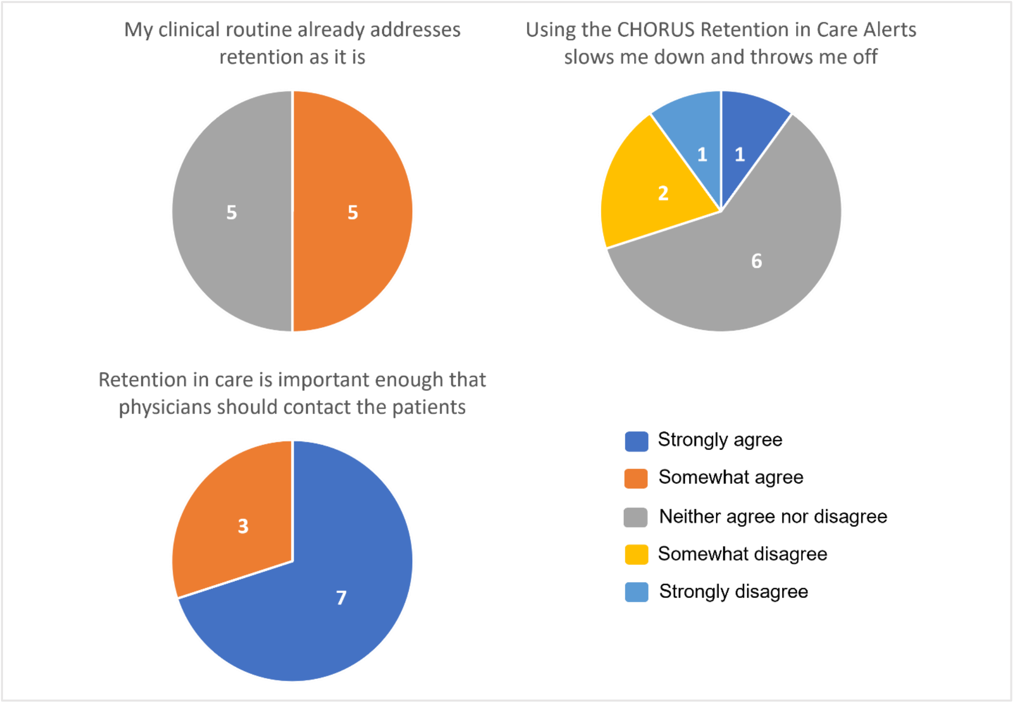

Additional Figure 2. Results of After First Use Survey: Adoption Domain Questions

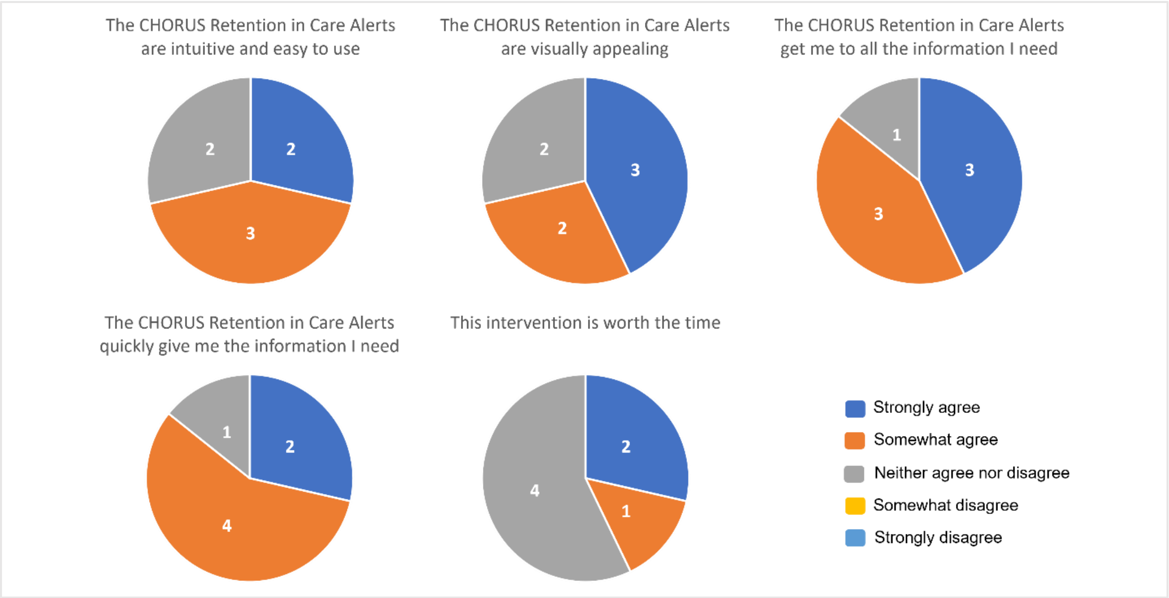

Additional Figure 3. Results of After 30 Days Survey: Acceptability Domain Questions

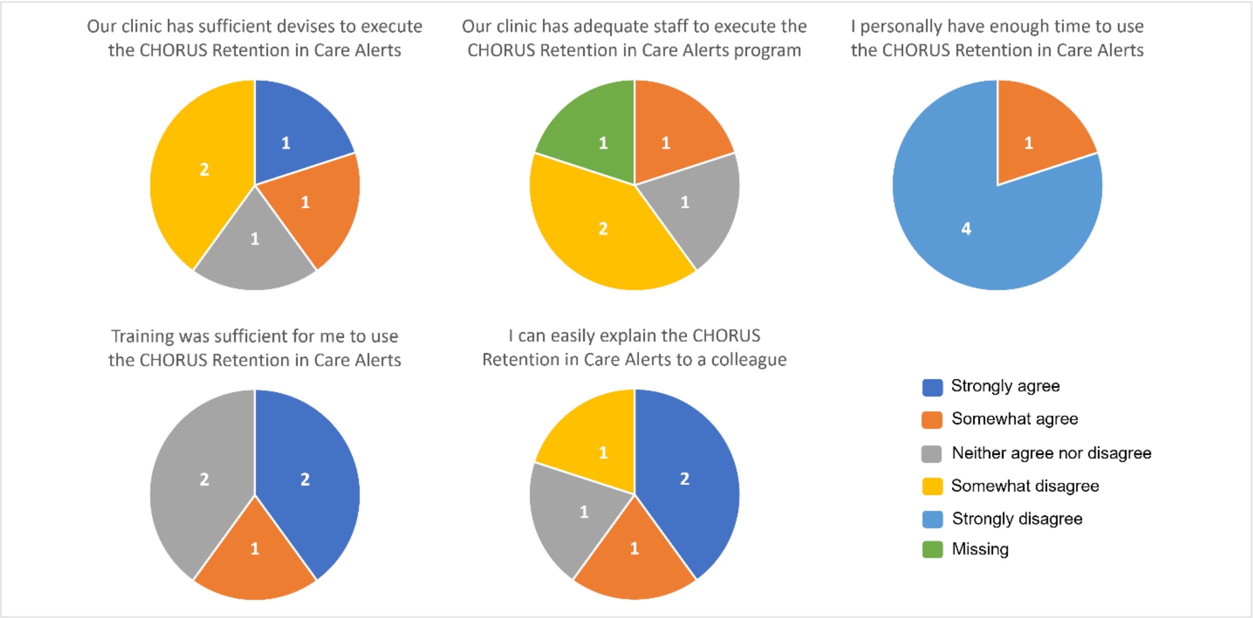

Additional Figure 4. Results of After 3 Months Survey: Feasibility Domain Questions

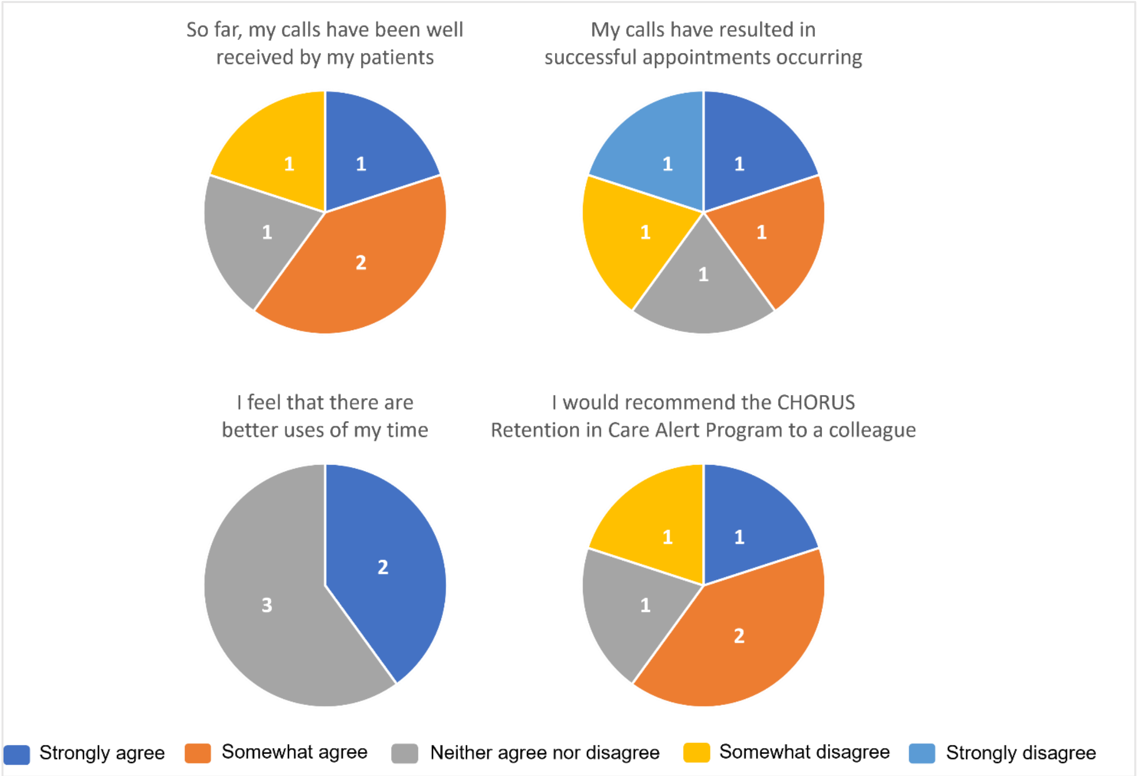

Additional Figure 5. Results of After 3 Months Survey: Fidelity Domain Questions

Feedback Received via Focus Groups

Representatives from 7 intervention healthcare centers (HCCs) and an HCC that participated in pilot activities provided feedback during two focus groups in July 2021 just prior to study completion. Comments were summarized and loosely assigned themes; they are further summarized below in **Additional Table 2**.

Additional Table 2. Summary of Comments Received During July 2021 Focus Groups, Grouped by Theme

Theme	Examples of Feedback Received
**Alerts**	
Specific alerts	Alerts 3 & 4 were too restrictive[a, b]
Requested alerts	Alert for any new patient who misses appointment in the first year
	Alerts on completed visits
Re-alerting	We see the same alerts for the same patients, month after month
	Adjust alerts for a longer time period before they become a new alert
Resolution of alerts	Some visits (labs, refills, Medicare wellness) should not count as a completed visit
**Comparison to Existing AHF Retention Efforts**	
Overlap	Concerned about patients on 104-day report who are not flagged by CHORUS™

Theme	Examples of Feedback Received
	Concern that CHORUS™ doesn't require provider engagement like 104-day report
	Can reports be merged so the CHORUS™ alerts highlight top priority patients?
	Would like to not have to duplicate retention notes in EHR and CHORUS™
	This process needs to be more efficient; AHF is asking HCC to work another report, without eliminating duplicative efforts
	The CHORUS™ RIC Module should help minimize the 104-day report
Preference	CHORUS™ is more accurate than the 104-day report
	Prefers the CHORUS™ alert system to monthly printouts from 104-day report
**Usability of CHORUS™ Retention in Care Module**	
Contacting Patient	Add option to call directly from the mobile app[c]
	Add option to have the call number blinded to stop patients from seeing the caller[d]
	Would like to contact patients by text/email/call directly from CHORUS™
Searching	Request to filter list by payer (e.g., Ryan White Program, commercial insurance)
	Request for searchable or alphabetized list
User experience	Likes how CHORUS™ looks
	Likes the CHORUS™ RIC Module and finds it easy to use
	Finds the CHORUS™ RIC Module cumbersome
**Incorporating CHORUS RIC Module into Clinic Workflow**	
Staff roles	Accessing CHORUS™ by staff members who are covering for others is a challenge
	Clinic staff members should have clearer responsibilities
Time	Running a clinic and making calls to patients is a challenge
	Our HCC took the "divide and conquer" approach to outreach
Challenge of EHR	Providers cannot schedule their own appointments in the EHR system
**Electronic Health Record Data**	
Accuracy	Discovered a lot of disconnected phone numbers
	Some patients trigger alerts even after they have transferred care
	Highlighted that some patients weren't assigned to the correct HCC in the EHR
Data refresh	Daily data refreshes were improvement over weekly refreshes earlier in the study
	Would prefer real-time data updates between EHR and CHORUS™
Relationship between EHR and CHORUS™	Build the CHORUS™ system directly into the EHR for efficiency
	Have data flow from CHORUS™ into the EHR
**General Feedback on the Positive Pathways Study**	
Value	Biggest value of the study was to change the "one and done" mindset of sites that would make just one call to the patient and give up. People now realize it takes multiple calls – sometimes from multiple people – to keep patients engaged.
	HCC feels they have made slow and steady progress
Unintended consequences	Suspects that some patients have blocked the clinic due to the number of phone calls
**General Feedback on CHORUS™**	
Overall	CHORUS™ helps improve the relationship between healthcare provider and patient
Other features	Quality care gaps in CHORUS™ are also helpful

EHR, electronic health record; HCC, healthcare center

[a] As of April 2021, the acceptable window for a subsequent appointment was moved from 7 days to 14 days following discussions with AHF retention leadership, due to the impracticality of scheduling patients within 7 days

[b] As of April 2021, the threshold for undetectability for Alert 4 was changed from < 20 copies/mL (i.e., test's limit of detection) to < 50 copies/mL to better reflect standard of care

[c] The CHORUS™ mobile app included this feature

[d] This feature was added to the CHORUS™ mobile app over the course of the study; AHF workforce additionally identified a workaround in their system that blinded the phone number
